# First case of mixed infection with *Cryptococcus deuterogattii* and *Cryptococcus neoformans* VNI in an Ivorian HIV-positive patient

**DOI:** 10.1099/jmmcr.0.005037

**Published:** 2016-08-30

**Authors:** Fulgence K. Kassi, Virginie Bellet, Adama Doumbia, Donika Krasteva, Pascal Drakulovski, Gisèle A. Kouakou, François Gatchitch, Eric Delaporte, Jacques Reynes, Michèle Mallié, Hervé I. E. Menan, Sébastien Bertout

**Affiliations:** ^1^​ Université Félix Houphouët Boigny, UFR Pharmacie, Laboratoire de Parasitologie et Mycologie – CeDReS (Centre de Diagnostic et de Recherche sur le SIDA et les autres maladies infectieuses), CHU de Treichville, BP V3, Abidjan, Cote d'Ivoire; ^2^​ UMI 233 IRD-UM-INSERM U1175 Laboratoire de Parasitologie et Mycologie médicale UFR Pharmacie, 15 Av. C. Flahault, BP 14491, 34093 Montpellier Cedex 5, France; ^3^​ Service des Maladies Infectieuses et Tropicales, CHU de Treichville, 01 BP V3, Abidjan, Côte d'Ivoire; ^4^​ UMI 233 Service des Maladies Infectieuses et Tropicales, CHU Gui de Chauliac`, Montpellier, France

**Keywords:** AIDS, *Cryptococcus deuterogattii*, * Cryptococcus neoformans*, genotyping, mixed infection, antifungal susceptibility

## Abstract

**Introduction:**

Cryptococcal meningitis (CM) may be caused by several species of *Cryptococcus. *

**Case presentation:**

We describe a fatal case of CM in a HIV-positive patient from Ivory Coast infected by *Cryptococcus neoformans* VNI and *Cryptococcus deuterogattii*. Isolates were recovered from cerebrospinal fluid (CSF) prior to systemic antifungal treatment. Six isolates were studied (the entire culture plus five isolated colonies from it). Serotyping was performed via LAC 1 and CAP 64 gene amplification. Genotyping was performed using restriction fragment length polymorphism (RFLP) analysis of the URA5 gene, (GACA)_4_, (GTG)_5 _and M13 PCR fingerprinting. URA5-RFLP analysis identified the original culture with two different molecular type combinations. However, URA5-RFLP profiles of the five colonies isolated from the original sample revealed two different species. Four colonies were identified as *C. deuterogattii* and the last isolate as *C. neoformans* VNI. The *in vitro* susceptibility profile was determined using the standard method according to the CLSI M27-A3 protocol. The isolates were susceptible to the tested antifungals (fluconazole, flucytosine and amphotericin B). Treatment with fluconazole (1200  mg day^−1^) was initiated; however, the patient died 17 days after the onset of antifungal therapy.

**Conclusion:**

This is the first reported case of mixed infection with *C. neoformans* and *C. deuterogattii* in a HIV-positive patient.

## Introduction

*Cryptococcus *complex species is a major cause of fungal meningoencephalitis in immunocompromised patients in Africa (Aoussi *et al*., 2012; Bertout *et al*., 2013). This encapsulated basidiomycetous yeast is widespread in the environment, where it is associated most commonly with avian excreta and tree hollows (Connolly *et al*., 1999). The initial infection is acquired by inhalation of fungal cells from an environmental source where mixed yeast populations may co-exist. The taxonomy of the *C*ryptococcus* neoformans*/*C*ryptococcus* gattii *complex species has recently been revised and contains seven proposed species and thirteen genotypes (Hagen *et al.,* 2015). A single isolate of *Cryptococcus *complex species has been thought to be responsible for the disease (Desnos-Ollivier *et al*., 2010). However, the isolation of strains with different genotypes, serotypes or species during the same episode in a unique sample makes infestation by multiple strains likely (Aminnejad *et al*., 2012; Barchiesi* et al*., 1995; Bertout *et al*., 2013; Bovers *et al*., 2006, 2008; Igreja *et al*., 2004; Illnait-Zaragozí* et al*., 2010; Mlinaric-Missoni *et al*., 2011). Isolates are usually susceptible to the standard clinically used antifungals , including fluconazole, 5-fluorocytosine and amphotericin B (Mlinaric-Missoni *et al*., 2011; Favalessa* et al*., 2014). The use of antifungal agents, particularly in long-term suppressive regimens, has raised concern about the development of drug resistance in *C. neoformans. *Resistance to antifungal drugs is scarce among clinical isolates of *C. neoformans* but has been reported. Nevertheless, reduced susceptibility between different species of *Cryptococcus* has been demonstrated worldwide (Chong *et al.,* 2010; Chowdhary *et al.,* 2011; Trilles *et al.,* 2012).

We describe the first report of mixed infection with *C. neoformans* VNI and *C. deuterogattii* in a HIV-positive patient. The possibility of mixed infection must be considered for the management of cryptococcosis.

## Case report

In January 2014, a 41-year-old male was hospitalized in the Infectious and Tropical Diseases Unit of Treichville (Abidjan, Ivory Coast) because of complaints of high-grade fever, and severe headache lasting for three weeks associated with depressed level of consciousness and multiple episodes of vomiting.

Known to be HIV-1 positive since 2012, this patient was under second line of antiretroviral treatment combining tenofovir (245 mg day^−^^1^), emtricitabine (200 mg day^−^^1^) and lopinavir/ritonavir (400/100 mg twice daily).

## Investigations

On physical examination, the patient was confused and his speech was incoherent. No meningismus or focal neurological signs were found. Full blood count revealed a decrease of haemoglobin down to 7.6 g  dl^−1^, CD4 count was 75 cells mm^−^^3^ and the plasma HIV viral load was 704 275 copies ml^−1^. Lumbar puncture revealed an elevated opening pressure (40 cm H_2_O) of the cerebrospinal fluid (CSF) containing a low glucose concentration (0.17 g l^−^^1^) and elevated protein level (1.35 g l^−^^1^). CSF bacterial cultures were sterile. However, direct microscopic examination of the CSF using India ink detected numerous encapsulated yeasts suggesting *Cryptococcus spp.* and a diagnosis of cryptococcal meningitis (CM) was given by the physicians.

## Diagnosis

Culture of the CSF on Sabouraud dextrose agar medium (Biomerieux) at 37 °C for 3 days allowed isolation of smooth yellowish colonies that subsequently showed to be urease-positive. A positive test was observed by a colour change from orange to pink, due to the production of urease by *Cryptococcus* complex species in the medium. For urease-positive cultures, culture on Niger seeds agar, as previously described, was carried out to certify that the strains present are effectively of *Cryptococcus* species (Staib *et al*., 1987). Colonies of *Cryptococcus* complex species were identified by production of brown melanin pigment. Finally, the genus and the species were confirmed by using the API 20C test (Biomerieux).

Phenotypic characterization of the *Cryptococcus* species was achieved by chemotyping in l-canavanine-glycine-bromothymol blue (CGB) agar. CGB agar was used to differentiate *C. neoformans* complex species and *C. gattii *complex species as described previously (Klein *et al*., 2009). Growth of *C. gattii* complex species on CGB agar produced a blue color, indicating the assimilation of glycine, while *C. neoformans* complex species failed to cause a colour change. Six isolates (entire culture and five isolated colonies) were separated for further investigations. A blue-colour change on CGB agar suggesting *C. gattii* was observed for four out of five colonies. Serotyping was performed via LAC 1 and CAP 64 gene amplification. To gain more interpretation of the isolates profiles, genotyping was performed using M13, (GACA)_4_ and (GTG)_5_ primers and RFLP analysis of the URA5 gene. Mixed patterns of both *C. neoformans* VNI and *Cryptococcus deuterogattii* species were observed in the entire culture. Concerning the isolated colonies, one was identified as *C. neoformans* VNI and four as *C. deuterogattii.*

Susceptibilities to amphotericin B, flucytosine and fluconazole were tested by the broth microdilution method according to the M27-A3 CLSI protocol, 2008 (CLSI, 2008). For *Cryptococcus *species, clinical break-points have not been established by the CLSI, so we used epidemiological cut-off values as described previously (Espinel-Ingroff *et al*., 2012a, b; Pfaller *et al*., 2005): fluconazole and flucytosine, susceptible ≤8 µg ml^−1^; amphotericin B, susceptible ≤1 µg ml^−1^. All isolates exhibited a low MIC value to fluconazole (4 µg ml^−1^), flucytosine (2 µg ml^−1^) and amphotericin B (0.5 µg ml^−1^).

## Treatment

Patient received high oral dose of fluconazole (1200 mg day^−^^1^).

## Outcome and follow-up

On day 12 of admission, the patient continued to display altered mental status (GCS 6/15) with unresolved pyrexia, headaches and persistently low haemoglobin unresponsive to transfusion. He died at day 17 after the initiation of antifungal therapy.

## Discussion

Due to the importance of the *C. neoformans*/*C. gattii *species complex as human fungal pathogens, several research groups are currently focusing on the molecular determination of the number of genetically divergent subgroups within each species. The application of molecular methods, such as PCR fingerprinting, amplified fragment length polymorphism (AFLP) and, more recently, multi-locus sequence typing (MLST) has led to a better insight into the taxonomy and identification of the causative agents of cryptococcosis (Bovers *et al*., 2006, 2008). Advances in phylogenetic and genotypic studies now suggest seven species in the *C. gattii*/*C. neoformans* species complex. Interspecies hybrids between *C. gattii* and *C. neoformans* were also considered: *C. neoformans* var. *neoformans *AFLP2/VNIV × *C. gattii* AFLP4/VGI (serotype BD, genotype AFLP8), *C. neoformans* var. *grubii* AFLP1/VNI × *C. gattii* AFLP4/VGI (serotype AB, genotype AFLP9) and *C. neoformans* var. *grubii* AFLP1/VNI × *C. deuterogattii* AFLP6/VGII (serotype AB, genotype AFLP11) (Aminnejad *et al*., 2012; Bovers *et al*., 2006, 2008).

Previous worldwide studies reported a greater genetic diversity among cryptococcal isolates (Aminnejad *et al*., 2012; Bertout *et al*., 2013; Desnos-Ollivier *et al*., 2010). In the past, a single isolate of *Cryptococcus *complex species has been thought to be responsible for the disease (Desnos-Ollivier *et al*., 2010). Various combinations of strains with different genotypes of the same species were found during the same episode of cryptococcosis, sometimes in the same sample (Barchiesi* et al*., 1995; Bertout *et al*., 2013; Haynes* et al*., 1995; Igreja *et al*., 2004; Illnait -Zaragozí* et al*., 2010; Mlinaric-Missoni *et al*., 2011). These results provided evidence for mixed infection with genetically distinct strains acquired from the environment. Infrequently, different serotype or different C*ryptococcus *species have also been identified during the same episode of CM (Aminnejad *et al*., 2012; Bovers *et al*., 2006, 2008; Desnos-Ollivier *et al*., 2010).

**Fig. 1.**
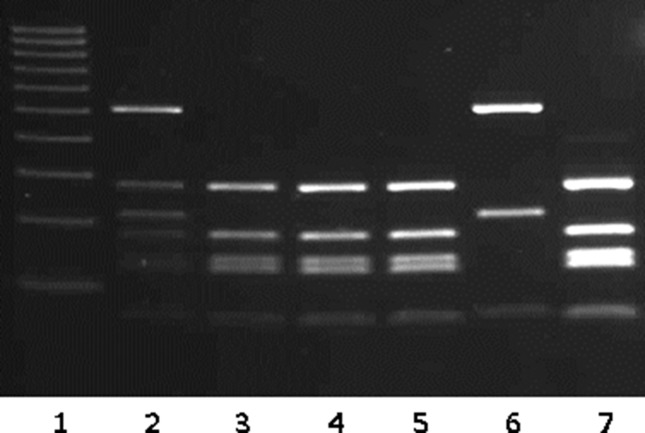
URA5 gene restriction fragment length polymorphism (RFLP) of the isolates. Lanes: 1, Ladder 100 pb (Sigma Aldrich); 2, entire culture; 3–7, the five isolated colonies with lanes 3, 4, 5 and 7 corresponding to *C. deuterogattii* profiles and lane 6 corresponding to *C. neoformans* VNI profile.

In the present case, serotyping performed on the culture was unable to identify a causative organism. URA5-RFLP analysis identified the original culture with two different molecular type combinations. The M13 PCR fingerprinting profile of this strain confirmed that the original sample contained fragments of both proposed parental groups (VNI and VGII). However, URA5-RFLP profiles of the five colonies isolated from the original sample revealed two different species (Fig. 1). Four colonies were identified as *C. deuterogattii* and the last isolate as *C. neoformans* VNI. To the best of our knowledge, our study is the first report in the literature documenting the occurrence of a mixed infection of both *C. deuterogattii* and *C. neoformans* VNI in a HIV- positive patient. In contrast, by using the same molecular typing technique, a previous study reported the occurrence of hybrids between *C. deuterogattii* and *C. neoformans* (Aminnejad *et al*., 2012). Until recently, *C. gattii *species complex was believed to primarily infect immunocompetent individuals, but our results and other reports demonstrated that immunocompromised patients could also be infected by these fungal species (Desnos-Ollivier *et al*., 2010; Illnait-Zaragozí* et al*., 2010; Mlinaric-Missoni *et al*., 2011).

It is known that exposure to environmental sources, such as contaminated trees and soil can lead to cryptococcosis infection in humans and animals. Recently, a new natural habitat for* C. neoformans* strains was described in Brazil, consisting of hollow trees in which both varieties of the yeast may be found simultaneously (Lazera *et al*., 2000). An *in vivo* study described the isolation of two varieties of *C. neoformans* from the upper respiratory tract of a koala, indicating a mixed colonization (Connolly *et al*., 1999). Our finding suggested the existence of environmental *C. deuterogattii* and *C. neoformans* species in Ivory Coast. Future studies will focus on recovery of *Cryptococcus* from soil, eucalyptus trees, flowers and pigeon droppings near human habitats. Understanding the environmental source of infection is critical for understanding the ecology and pathogenesis of this fungus.

Concerning the antifungal susceptibility, isolates of *C. neoformans* AFLP1/VNI were described in literature to have a significantly higher geometric mean MIC for fluconazole than isolates of the two subgenotypes AFLP1A/VNB/VNII and AFLP1B/VNII (Chong *et al.,* 2010). *C. neoformans* has been observed to be more susceptible to several antifungals when compared to *C. gattii* (Chowdhary *et al.,* 2011) and *C. deuterogattii* (Trilles *et al.,* 2012), but less susceptible for 5-fluorocytosine and amphotericin B when compared to *C. gattii* (Chau *et al.,* 2010). In our study, results showed that the two isolated species were susceptible to fluconazole, flucytosine and amphotericin B.

In Ivory Coast, despite the availability of antiretroviral treatment, most patients admitted for CM consult at late stages of the disease (Aoussi *et al*., 2012). The delay for consultation is even longer in cases of CM in HIV-negative patients because of the insidious evolution of symptoms reported in most series (Favalessa* et al*., 2014; Mlinaric-Missoni *et al*., 2011). At the time of admission, the patient presented severe headache, fever and vomiting. Previous studies have shown that headache is the predominant clinical sign of patients affected by *C. gattii* (Cookman *et al*., 2013; Favalessa* et al*., 2014, Steele *et al*., 2010).

Nevertheless, patients with mixed infections did not differ significantly from those with single infections in terms of underlying disease and clinical presentation (Desnos-Ollivier *et al*., 2010). For this case, the consultation at late stage, the severe immunodepression and the elevated CSF intracranial pressure could explain the early death of the patient.

This study is the first report on the presence of *C. neoformans* VNI and *C. deuterogattii* in a HIV-positive patient. It also highlights the first clinical isolation of *C. deuterogattii* from Ivory Coast.
